# Supplementary material for: Youth Positive Mental Health Concepts and Definitions: A Systematic Review and Qualitative Synthesis
Source: Int J Environ Res Public Health. 2022 Sep 13;19(18):11506. doi: 10.3390/ijerph191811506 (PMC9517177; doi:10.3390/ijerph191811506)
Supplement: Supplementary file 1 [file ijerph-19-11506-s001.zip › ijerph-1892000-supplementary.pdf]

Table S1: Detailed search terms used to identify articles

Pubmed	Additional Filters 1999 onwards, 'Humans', 'English'<br>full text search ((((((((((((((((((((mental wellbeing[Title/Abstract]) OR mental well-being[Title/Abstract]) OR mental well being[Title/Abstract]) OR mental wellness[Title/Abstract]) OR positive mental health[Title/Abstract]) OR psychological well-being[Title/Abstract]) OR psychological wellbeing[Title/Abstract]) OR psychological well being[Title/Abstract]) OR subjective well-being[Title/Abstract]) OR subjective well being[Title/Abstract]) OR subjective wellbeing[Title/Abstract]) OR self-determination theory[Title/Abstract]) OR happiness[Title/Abstract]) OR life satisfaction[Title/Abstract]) OR hierarchy of needs[Title/Abstract]) OR self-actualization[Title/Abstract]) OR big five personalit*[Title/Abstract]) OR big five trait*[Title/Abstract])) AND (((((((youth*[Title/Abstract]) OR adolescen*[Title/Abstract]) OR young adult*[Title/Abstract]) OR teen*[Title/Abstract]) OR young people[Title/Abstract]) OR student*[Title/Abstract]) OR adolescent[MeSH Terms])) AND ((((((((conceptual framework[Title/Abstract]) OR theoretical framework[Title/Abstract]) OR concept[Title/Abstract]) OR theory[Title/Abstract]) OR hypothesis[Title/Abstract]) OR psychological assessment[Title/Abstract]) OR psychological measurement[Title/Abstract]) OR psychological model[MeSH Terms])
Embase	Additional Filters 1999 onwards, 'Humans', 'English'<br>full text search ('conceptual framework'/exp OR 'conceptual framework':ab,ti OR 'theory':ab,ti OR 'hypothesis':ab,ti OR 'theoretical framework':ab,ti OR 'concept':ab,ti OR 'psychological assessment':ab,ti OR 'psychological measurement':ab,ti) AND [humans]/lim AND [english]/lim AND [1999-2019]/py<br>('adolescence'/exp OR 'youth*':ab,ti OR 'adolescen*':ab,ti OR 'teen*':ab,ti OR 'young adult*':ab,ti OR 'student*':ab,ti OR 'young people':ab,ti) AND [humans]/lim AND [english]/lim AND [1999-2019]/py<br>('psychological wellbeing':ab,ti OR 'psychological well being':ab,ti OR 'mental wellbeing':ab,ti OR 'mental well-being':ab,ti OR 'mental well being':ab,ti OR 'psychological well-being':ab,ti OR 'mental wellness':ab,ti OR 'positive mental health':ab,ti OR 'subjective wellbeing':ab,ti OR 'subjective well being':ab,ti OR 'subjective well-being':ab,ti OR 'self-determination theory':ab,ti OR 'hierarchy of needs':ab,ti OR 'happiness':ab,ti OR 'life satisfaction':ab,ti OR 'big five personalit*':ab,ti OR 'big five trait*':ab,ti OR 'self-actualization':ab,ti) AND [humans]/lim AND [english]/lim AND [1999-2019]/py
PsycInfo	Additional Filters 1999 onwards, 'Humans', 'English'<br>full text search (conceptual framework or hypothesis or theory or theoretical framework or concept or psychological assessment or psychological measurement).ab. or psychological model.mh.<br>(youth* or adolescen* or teen* or young adult* or young people or student*).ab. or adolescent.mh.

	(mental well-being or mental well being or mental well-being or mental wellness or positive mental health or psychological well-being or psychological wellbeing or psychological well being or subjective well-being or subjective wellbeing or subjective well being or self-determination theory or hierarchy of needs or happiness or life satisfaction or self-actualization or big five personalit* or big five trait*).ab.
OpenGrey	Additional Filters 1999 onwards, 'Humans', 'English'<br>full text search (abstract:"mental well being" OR abstract:"psychological well being" OR abstract:"psychological well-being" OR abstract:"mental wellness" OR abstract:"positive mental health" OR abstract:"psychological wellbeing" OR abstract:"subjective wellbeing" OR abstract:"subjective well being" OR abstract:"subjective well-being" OR abstract:"happiness" OR abstract:"self-determination theory" OR abstract:"hierarchy of needs" OR abstract:"self-actualization" OR abstract:big five personalit* OR abstract:big five trait*) AND (abstract:youth* OR abstract:adolescen* OR abstract:teen* OR abstract:young adult* OR abstract:"young people" OR abstract:student*) AND (abstract:"conceptual framework" OR abstract:"theory" OR abstract:"hypothesis" OR abstract:"theoretical framework" OR abstract:"concept" OR abstract:"psychological assessment" OR abstract:"psychological measurement")

Table S2: Characteristics of included studies (N=105)

No	Citation	Region	Study population	Age	N
1	Arce *et al.*, 2009 [80]	North America	University students	17-26, m18	65
2	Archana *et al.*, 2014 [81]	Asia	University students	21-24	186
3	Baldwin & Caldwell, 2003 [78]	North America	Middle/High school students	NS, grade 7	634
4	Barbot *et al.*, 2019 [48]	Europe	Middle/High school students	13-19	2603
5	Barry, 2015 [106]	North America	Middle/High school students	NS, 10-12th grade	15
6	Beduna & Perrone-McGoven, 2016, 2016 [92]	North America	University students	18-25	144
7	Benita *et al.*, 2017 [87]	Asia	Secondary school students	m12.5,	
8	Berzonsky & Huprich, 2016 [25]	Europe	University students	18-22	654
9	Bhattacharya, 2011 [68]	Asia	University students	23-28	10
10	Bieg *et al.*, 2017 [57]	Europe	University students	m21	1156
11	Bjorck *et al.*, 2019 [79]	North America	Secondary school students	11-19,	523
12	Bond *et al.*, 2014 [115]	Australia	Secondary school students	13-14	120
13	Bornstein & Huprich, 2006 [101]	North America	University students	m18.5	192
14	Bourke & Geldens, 2007 [111]	Australia	Secondary school students	16-24	19
15	Bradley *et al.*, 2014 [108]	North America	Community	m15	965
16	Burns & D'Zurilla, 1999 [85]	North America	University students	m21	1265
17	Caprara *et al.*, 2012 [121]	Europe, North America, Asia	Community	m21	7741
18	Caprara *et al.*, 2006 [44]	Europe	Middle/High school students	m16	664
19	Cheng, 2004 [91]	Asia	University students	m22	75
20	Cohn *et al.*, 2009 [82]	North America	University students	m18	86
21	Chinekesh *et al.*, 2018 [96]	Asia	Community	18-30	21
22	Cooper et al., 2015 [26]	North America	Community	m21	446
23	Datu, 2013 [46]	Asia	University students	15-18, m17	470
24	Davids *et al.*, 2017 [59]	South Africa	Secondary school students	m16	457
25	Demyttenaere *et al.*, 2019 [89]	Europe	University students	NS	203
26	Don, 2016 [66]	Europe	University students	18-28, m 19	138

27	Du *et al.*, 2017 [43]	Asia	University students	m18	847
28	Faye & Sharpe, 2008 [31]	North America	University students	18-25, m19	362
29	Fernandes *et al.*, 2010 [116]	Europe	Secondary school students	10-29, m14	1963
30	Furlong *et al.*, 2014 [88]	North America	Middle/High school students	13-18, m15	4189
31	Gao *et al.*, 2018 [117]	Asia	Middle/High school students	12-15, m13	365
32	Gilman *et al.*, 2003 [109]	NA	Community	NA	NA
33	Gomez-Baya *et al.*, 2018 [93]	Europe	Secondary school students	13-16, m13	977
34	Gongora & Solano, 2014 [67]	South America	Middle/High school students	13-18, m15	255
35	Gongora & Solano, 2015 [73]	South America	Middle/High school students	13-18, m15	255
36	Gruenewald, 2010 [32]	North America, Asia	University students	m21 apporx.	504
37	Hall *et al.*, 2015 [69]	NA	Community	12-35,	
38	Hardin & Larsen, 2014 [45]	North America	University students	m18	641
39	Hatcher & Rogers, 2009 [102]	North America	University students	18-26, m19	2490
40	Hathcoat, 2014 [20]	North America	University students	m19	683
41	Hathcoat & Fuqua, 2014 [23]	North America	University students	17-52, m20	834
42	Hitokoto & Uchida, 2015 [39]	North America, Asia	University students	m19	605
43	Hui & Tsang, 2012 [17]	NA	Community	NA	NA
44	Huo *et al.*, 2010 [77]	North America	Middle/High school students	m15	1377
45	Isik *et al.*, 2018 [61]	Asia	University students	m19	303
46	Janke & Dickhauser, 2019 [54]	Europe	University students	m23.5, 20	510
47	Kelley *et al.*, 2000 [14]	Europe, North America, Asia	At-risk adolescents	14-20	1892
48	Kern *et al.*, 2016 [53]	North America, Australia	Community	11-19,	4480
49	Kern *et al.*, 2015 [62]	Australia	University students	13-18	516
50	Kim *et al.*, 2003 [37]	North America, Asia	University students	NS	537
51	Kindelberger & Tsao, 2014 [104]	Europe	Middle/High school students	14-19	284
52	Kitayama *et al.*, 2000 [95]	North America, Asia	University students	NS	913

53	Kitayama *et al.*, 2009 [38]	Europe, North America, Asia	University students	m20	707
54	Krems *et al.*, 2017 [52]	North America	University students	18-36,m19	208
55	Kuo *et al.*, 2018 [40]	North America	University students	m21	328
56	La Guardia *et al.*, 2000 [105]	North America	University students	NS	448
57	Lambert D'raven & Pasha-Zaidi, 2016 [65]	Asia	University students	16-31,m19	54
58	Landstedt *et al.*, 2009 [63]	Europe	Middle/High school students	16-19	29
59	Lane & Mathes, 2018 [71]	North America	University students	m19	383
60	Lau *et al.*, 2019 [86]	Asia	University students	m20	1530
61	Leak *et al.*, 2007 [60]	North America	University students	17-38, m19	134
62	Lens *et al.*, 2012 [51]	NA	Community	NA	NA
63	Leon & Nunez, 2013 [30]	Europe	University students	m21	272
64	Li *et al.*, 2011 [103]	Asia	Community	17-22	96
65	Long *et al.*, 2012 [112]	Asia	Secondary school students	11-18, m15	1476
66	Mahon & Yarcheski, 2002 [50]	North America	Middle/High school students	12-14, m13	160
67	Maltby *et al.*, 1999 [120]	Europe	University students	18-29,m20	474
68	McCullough *et al.*, 2000 [36]	North America	Middle/High school students	NS, grade 9-12	92
69	Michou *et al.*, 2016 [58]	Asia	University students	m20	557
70	Moneta, 2004 [74]	North America, Asia	University students	18-23, m19	829
71	Mouratidis & Michou, 2011 [24]	Europe	Secondary school students	12-12,	426
72	Muenks *et al.*, 2018 [42]	NA	Community	NA	NA
73	Norrish & Vella-Brodrick, 2009 [56]	NA	Community	NA	NA
74	Nott & Vuchinich, 2016 [12]	North America	Homeless adolescents	12-17,	38
75	Oberle *et al.*, 2011 [107]	North America	Secondary school students	m11	1402
76	O'Higgins *et al.*, 2010 [97]	Europe	Secondary school students	12-13,	31
77	Palomar-Lever & Victorio-Estrada, 2014 [113]	South America	Community	12-19, m14	1093
78	Parker *et al.*, 2008 [41]	Australia	University students	mostly 18-25, m22	523
79	Patrick *et al.*, 2007 [19]	North America	Community	18-55, m23	2040
80	Pilarska, 2018 [47]	Europe	University students	18-31, m21	357

81	Renshaw *et al.*, 2015 [72]	North America	Middle/High school students	NS, grade 6-8	1002
82	Renshaw & Olinger Steeves, 2016 [90]	NA	Community	NA	NA
83	Robak *et al.*, 2011 [132]	North America	University students	18-39	449
84	Rodriguez-Fernandez *et al.*, 2016a [35]	Europe	Secondary school students	12-15, m13	1250
85	Rodriguez-Fernandez *et al.*, 2016b [76]	Europe	Secondary school students	12-15, m13	1250
86	Roldan-Merino *et al.*, 2017 [29]	Europe	University students	18-56, m21	1091
87	Sheldon & Hilpert, 2012 [27]	North America	University students	NS	324
88	Sheldon & Lyubomirsky, 2012 [64]	North America	University students	NS	481
89	Sheldon *et al.*, 2017 [28]	North America, Europe	University students	m19	1547
90	Shoshana, 2019 [94]	Asia	Middle/High school students	16-18	36
91	Smith *et al.*, 2017 [118]	Europe	Middle/High school students	15	2217
92	Soenens *et al.*, 2011 [33]	North America	University students	12-25, m19	431
93	Usborne *et al.*, 2009 [13]	North America	Street kids	15-26, m20	50
94	Vainio & Daukantaite, 2016 [34]	Europe	University students	m26	592
95	Valle *et al.*, 2006 [55]	North America	University students	10-18,m13	860
96	Van Ryzin *et al.*, 2009 [21]	North America	Secondary school students	m15	283
97	Van Schaick, 2011 [83]	North America	University students	18-21, m18	431
98	Vitterso, 2004 [75]	Europe	Middle/High school students	16-25	264
99	Walker, 2015 [18]	North America	Community	16-21	130
100	Wei *et al.*, 2011 [114]	North America	University students	18-42, m20	195
101	Weigold *et al.*, 2013 [49]	North America	University students	18-44, m21	331
102	Weinstein & Ryan, 2010 [22]	North America	University students	18-25	218
103	Wright *et al.*, 2017 [84]	North America	University students	m19	538
104	Yang *et al.*, 2017 [70]	Asia	Middle/High school students	13-18	2082
105	Zou *et al.*, 2013 [110]	North America	University students	17-30, m19	335

m: mean age of the sample; NS: not specified

References

1. Maslow, A.H. A theory of human motivation. *Psychol. Rev.* **1943**, *50*, 370–396. https://doi.org/10.1037/h0054346.
2. Noltemeyer, A.; Bush, K.; Patton, J.; Bergen, D. The relationship among deficiency needs and growth needs: An empirical investigation of Maslow's theory. *Child. Youth Serv. Rev.* **2012**, *34*, 1862–1867. https://doi.org/10.1016/j.childyouth.2012.05.021.
3. Jahoda, M. *Current Concepts of Positive Mental Health*; Basic Books: New York, NY, US, 1958.
4. Ryan, R.M.; Deci, E.L. On happiness and human potentials: A review of research on hedonic and eudaimonic well-being. *Annu. Rev. Psychol.* **2001**, *52*, 141–166. https://doi.org/10.1146/annurev.psych.52.1.141.
5. Tang, M.; Wang, D.; Guerrien, A. A systematic review and meta-analysis on basic psychological need satisfaction, motivation, and well-being in later life: Contributions of self-determination theory. *PsyCh J.* **2020**, *9*, 5–33. https://doi.org/10.1002/pchj.293.
6. Organization, W.H. *Promoting Mental Health: Concepts, Emerging Evidence, Practice: Summary Report*; World Health Organization: Geneva, Switzerland, 2004.
7. Nations, U. Global Issues: Youth. Available online: https://www.un.org/en/global-issues/youth#:~:text=There%20is%20no%20universally%20agreed,of%2015%20and%2024%20years (accessed on 5 December 2021).
8. Council, N.Y. Youth Scope Report. Available online: https://www.youthpolicy.org/library/wp-content/uploads/library/Singapore_2012_Youth_Scope_Report_eng.pdf (accessed on 5 December 2021).
9. Harden, A.; Garcia, J.; Oliver, S.; Rees, R.; Shepherd, J.; Brunton, G.; Oakley, A. Applying systematic review methods to studies of people's views: An example from public health research. *J. Epidemiol. Community Health* **2004**, *58*, 794–800. https://doi.org/10.1136/jech.2003.014829.
10. Tofthagen, R.; Fagerstrom, L.M. Rodgers' evolutionary concept analysis—A valid method for developing knowledge in nursing science. *Scand. J. Caring Sci.* **2010**, *24*, 21–31. https://doi.org/10.1111/j.1471-6712.2010.00845.x.
11. Bearman, M.; Dawson, P. Qualitative synthesis and systematic review in health professions education. *Med. Educ.* **2013**, *47*, 252260. https://doi.org/10.1111/medu.12092.
12. Nott, B.D.; Vuchinich, S. Homeless adolescents' perceptions of positive development: A comparative study. *Child Youth Care Forum* **2016**, *45*, 865–886.
13. Usborne, E.; Lydon, J.E.; Taylor, D.M. Goals and social relationships: Windows into the motivation and well-being of "street kids.". *J. Appl. Soc. Psychol.* **2009**, *39*, 1057–1082.
14. Kelley, T.M.; Stack, S.A. Thought recognition, locus of control, and adolescent well-being. *Adolescence* **2000**, *35*, 531–550.
15. Diener, E. Subjective well-being. The science of happiness and a proposal for a national index. *Am. Psychol.* **2000**, *55*, 34–43.
16. Ryff, C.D. Happiness is everything, or is it? Explorations on the meaning of psychological well-being. *J. Personal. Soc. Psychol.* **1989**, *57*, 1069–1081. https://doi.org/10.1037/0022-3514.57.6.1069.
17. Hui, E.K.; Tsang, S.K. Self-determination as a psychological and positive youth development construct. *Sci. World J.* **2012**, *2012*, 759358. https://doi.org/10.1100/2012/759358.
18. Walker, A.S. Understanding the relationship between family environment and psychological well-being in disconnected emerging adults: A mixed methods approach. *Diss. Abstr. Int. Sect. B: Sci. Eng.* **2015**, *75*.
19. Patrick, H.; Knee, C.R.; Canevello, A.; Lonsbary, C. The role of need fulfillment in relationship functioning and well-being: A self-determination theory perspective. *J. Pers. Soc. Psychol.* **2007**, *92*, 434–457. https://doi.org/10.1037/0022-3514.92.3.434.
20. Hathcoat, J.D. Categorical organization of numinous constructs, basic psychological need fulfillment, and psychological outcomes: A moderated mediation model. *Diss. Abstr. Int. Sect. B Sci. Eng.* **2014**, *74*.
21. Van Ryzin, M.J.; Gravely, A.A.; Roseth, C.J. Autonomy, belongingness, and engagement in school as contributors to adolescent psychological well-being. *J. Youth Adolesc.* **2009**, *38*, 1–12. https://doi.org/10.1007/s10964-007-9257-4.
22. Weinstein, N.; Ryan, R.M. When helping helps: Autonomous motivation for prosocial behavior and its influence on well-being for the helper and recipient. *J. Personal. Soc. Psychol.* **2010**, *98*, 222–244.
23. Hathcoat, J.D.; Fuqua, D.R. Initial development and validation of the basic psychological needs questionnaire-religiosity/spirituality. *Psychol. Relig. Spiritual.* **2014**, *6*, 53–63.
24. Mouratidis, A.; Michou, A. Self-determined motivation and social achievement goals in children's emotions. *Educ. Psychol.* **2011**, *31*, 67–86.
25. Berzonsky, M.D.; Cieciuch, J. Mediational role of identity commitment in relationships between identity processing style and psychological well-being. *J. Happiness Stud.* **2014**, *17*, 145–162. https://doi.org/10.1007/s10902-014-9588-2.
26. Cooper, S.; Lavaysse, L.M.; Gard, D.E. Assessing motivation orientations in schizophrenia: Scale development and validation. *Psychiatry Res.* **2015**, *225*, 70–78. https://doi.org/10.1016/j.psychres.2014.10.013.

27. Sheldon, K.M.; Hilpert, J.C. The Balanced Measure of Psychological Needs (BMPN) scale: An alternative domain general measure of need satisfaction. *Motiv. Emot.* **2012**, *36*, 439–451.
28. Sheldon, K.M.; Osin, E.N.; Gordeeva, T.O.; Suchkov, D.D.; Sychev, O.A. Evaluating the dimensionality of self-determination theory's relative autonomy continuum. *Pers. Soc. Psychol. Bull.* **2017**, *43*, 1215–1238. https://doi.org/10.1177/0146167217711915.
29. Roldan-Merino, J.; Lluch-Canut, M.T.; Casas, I.; Sanroma-Ortiz, M.; Ferre-Grau, C.; Sequeira, C.; Falco-Pegueroles, A.; Soares, D.; Puig-Llobet, M. Reliability and validity of the positive mental health questionnaire in a sample of Spanish university students. *J. Psychiatr. Ment. Health. Nurs.* **2017**, *24*, 123–133. https://doi.org/10.1111/jpm.12358.
30. Leon, J.; Nunez, J.L. Causal ordering of basic psychological needs and well-being. *Soc. Indic. Res.* **2013**, *114*, 243–253.
31. Faye, C.; Sharpe, D. Academic motivation in university: The role of basic psychological needs and identity formation. *Can. J. Behav. Sci.* **2008**, *40*, 189–199.
32. Gruenewald, J.M. Self-determination theory and hedonic well-being in a cross-cultural perspective. *Diss. Abstr. Int. Sect. B Sci. Eng.* **2010**, *70*, 7899.
33. Soenens, B.; Berzonsky, M.D.; Dunkel, C.S.; Papini, D.R.; Vansteenkiste, M. Are all identity commitments created equally? The importance of motives for commitment for late adolescents' personal adjustment. *Int. J. Behav. Dev.* **2011**, *35*, 358–369. https://doi.org/10.1177/0165025411405954.
34. Vainio, M.M.; Daukantaite, D. Grit and different aspects of well-being: Direct and indirect relationships via sense of coherence and authenticity. *J. Happiness Stud. Interdiscip. Forum Subj. Well-Being* **2016**, *17*, 2119–2147.
35. Rodriguez-Fernandez, A.; Ramos-Diaz, E.; Fernandez-Zabala, A.; Goni, E.; Esnaola, I.; Goni, A. Contextual and psychological variables in a descriptive model of subjective well-being and school engagement. *Int. J. Clin. Health Psychol.* **2016**, *16*, 166–174. https://doi.org/10.1016/j.ijchp.2016.01.003.
36. McCullough, G.; Huebner, E.; Laughlin, J.E. Life events, self-concept, and adolescents' positive subjective well-being. *Psychol. Sch.* **2000**, *37*, 281–290.
37. Kim, Y.; Kasser, T.; Lee, H. Self-concept, aspirations, and well-being in South Korea and the United States. *J. Soc. Psychol.* **2003**, *143*, 277–290. https://doi.org/10.1080/00224540309598445.
38. Kitayama, S.; Park, H.; Sevincer, A.T.; Karasawa, M.; Uskul, A.K. A cultural task analysis of implicit independence: Comparing North America, Western Europe, and East Asia. *J. Pers. Soc. Psychol.* **2009**, *97*, 236–255. https://doi.org/10.1037/a0015999.
39. Hitokoto, H.; Uchida, Y. Interdependent happiness: Theoretical importance and measurement validity. *J. Happiness Stud. Interdiscip. Forum Subj. Well-Being* **2015**, *16*, 211–239.
40. Kuo, B.C.; Soucie, K.M.; Huang, S.; Laith, R. The mediating role of cultural coping behaviours on the relationships between academic stress and positive psychosocial well-being outcomes. *Int. J. Psychol.* **2018**, *53*, 27–36.
41. Parker, P.D.; Martin, A.J.; Marsh, H.W. Factors predicting life satisfaction: A process model of personality, multidimensional self-concept, and life satisfaction. *Aust. J. Guid. Couns.* **2008**, *18*, 15–29.
42. Muenks, K.; Wigfield, A.; Eccles, J.S. I can do this! The development and calibration of children's expectations for success and competence beliefs. *Dev. Rev.* **2018**, *48*, 24–39.
43. Du, H.; King, R.B.; Chi, P. Self-esteem and subjective well-being revisited: The roles of personal, relational, and collective self-esteem. *PLoS ONE* **2017**, *12*, e0183958. https://doi.org/10.1371/journal.pone.0183958.
44. Caprara, G.V.; Steca, P.; Gerbino, M.; Pacielloi, M.; Vecchio, G.M. Looking for adolescents' well-being: Self-efficacy beliefs as determinants of positive thinking and happiness. *Epidemiol. E Psichiatr. Soc.* **2006**, *15*, 30–43. https://doi.org/10.1017/s1121189x00002013.
45. Hardin, E.E.; Larsen, J.T. Distinct sources of self-discrepancies: Effects of being who you want to be and wanting to be who you are on well-being. *Emotion* **2014**, *14*, 214–226. https://doi.org/10.1037/a0033893.
46. Datu, J.A.D. Can Happiness Boost Self-worth?: Exploring the impact of subjective well-being on the global self-esteem of Filipino adolescents. *J. Asia Pac. Couns.* **2013**, *3*, 131–138. https://doi.org/10.18401/2013.3.2.2.
47. Pilarska, A. Big-Five personality and aspects of the self-concept: Variable- and person-centered approaches. *Personal. Individ. Differ.* **2018**, *127*, 107–113. https://doi.org/10.1016/j.paid.2018.01.049.
48. Barbot, B.; Safont-Mottay, C.; Oubrayrie-Roussel, N. Multidimensional scale of self-esteem (EMES-16): Psychometric evaluation of a domain-specific measure of self-esteem for French-speaking adolescents. *Int. J. Behav. Dev.* **2019**, *43*, 436–446. https://doi.org/10.1177/0165025418824996.
49. Weigold, I.K.; Porfeli, E.J.; Weigold, A. Examining tenets of personal growth initiative using the personal growth initiative scale-II. *Psychol. Assess.* **2013**, *25*, 1396–1403. https://doi.org/10.1037/a0034104.
50. Mahon, N.E.; Yarcheski, A. Alternative theories of happiness in early adolescents. *Clin. Nurs. Res.* **2002**, *11*, 306–323. https://doi.org/10.1177/10573802011003006.
51. Lens, W.; Paixao, M.P.; Herrera, D.; Grobler, A. Future time perspective as a motivational variable: Content and extension of future goals affect the quantity and quality of motivation. *Jpn. Psychol. Res.* **2012**, *54*, 321–333.

52. Krems, J.A.; Kenrick, D.T.; Neel, R. Individual perceptions of self-actualization: What functional motives are linked to fulfilling one's full potential? *Pers. Soc. Psychol. Bull.* **2017**, *43*, 1337–1352. https://doi.org/10.1177/0146167217713191.
53. Kern, M.L.; Benson, L.; Steinberg, E.A.; Steinberg, L. The EPOCH measure of adolescent well-being. *Psychol. Assess.* **2016**, *28*, 586–597. https://doi.org/10.1037/pas0000201.
54. Janke, S.; Dickhäuser, O. A neglected tenet of achievement goal theory: Associations between life aspirations and achievement goal orientations. *Personal. Individ. Differ.* **2019**, *142*, 90–99. https://doi.org/10.1016/j.paid.2019.01.038.
55. Valle, M.F.; Huebner, E.; Suldo, S.M. An analysis of hope as a psychological strength. *J. Sch. Psychol.* **2006**, *44*, 393–406.
56. Norrish, J.M.; Vella-Brodrick, D.A. Positive psychology and adolescents: Where are we now? Where to from here? *Aust. Psychol.* **2009**, *44*, 270–278. https://doi.org/10.1080/00050060902914103.
57. Bieg, S.; Reindl, M.; Dresel, M. The relation between mastery goals and intrinsic motivation among university students: A longitudinal study. *Educ. Psychol.* **2016**, *37*, 666–679. https://doi.org/10.1080/01443410.2016.1202403.
58. Michou, A.; Matos, L.; Gargurevich, R.; Gumus, B.; Herrera, D. Building on the enriched hierarchical model of achievement motivation: autonomous and controlling reasons underlying mastery goals. *Psychol. Belg.* **2016**, *56*, 269–287. https://doi.org/10.5334/pb.281.
59. Davids, E.L.; Roman, N.V.; Kerchhoff, L.J. Adolescent goals and aspirations in search of psychological well-being: From the perspective of self-determination theory. *South Afr. J. Psychol.* **2016**, *47*, 121–132. https://doi.org/10.1177/0081246316653744.
60. Leak, G.K.; DeNeve, K.M.; Greteman, A.J. The relationship between spirituality, assessed through self-transcendent goal strivings, and positive psychological attributes. *Res. Soc. Sci. Study Relig.* **2007**, *18*, 263–279.
61. Işık, E.; Ulubey, E.; Kozan, S. An examination of the social cognitive model of well-being in Turkish college students. *J. Vocat. Behav.* **2018**, *106*, 11–21. https://doi.org/10.1016/j.jvb.2017.11.010.
62. Kern, M.L.; Waters, L.E.; Adler, A.; White, M.A. A multidimensional approach to measuring well-being in students: Application of the PERMA framework. *J. Posit. Psychol.* **2015**, *10*, 262–271. https://doi.org/10.1080/17439760.2014.936962.
63. Landstedt, E.; Asplund, K.; Gillander Gadin, K. Understanding adolescent mental health: The influence of social processes, doing gender and gendered power relations. *Sociol. Health Illn.* **2009**, *31*, 962–978. https://doi.org/10.1111/j.1467-9566.2009.01170.x.
64. Sheldon, K.M.; Lyubomirsky, S. The challenge of staying happier: Testing the Hedonic Adaptation Prevention model. *Pers. Soc. Psychol. Bull.* **2012**, *38*, 670–680. https://doi.org/10.1177/0146167212436400.
65. Lambert D'raven, L.; Pasha-Zaidi, N. Using the PERMA model in the United Arab Emirates. *Soc. Indic. Res.* **2016**, *125*, 905–933.
66. Don, B.P. The influence of relationship closeness on the feeling that life is meaningful. *Diss. Abstr. Int. Sect. B Sci. Eng.* **2016**, *76*.
67. Góngora, V.C.; Castro Solano, A. Well-being and life satisfaction in Argentinean adolescents. *J. Youth Stud.* **2014**, *17*, 1277–1291. https://doi.org/10.1080/13676261.2014.918251.
68. Bhattacharya, A. Meaning in Life: A qualitative inquiry into the life of young adults. *Psychol. Stud.* **2011**, *56*, 280–288. https://doi.org/10.1007/s12646-011-0091-0.
69. Hall, S.; McKinstry, C.; Hyett, N. An occupational perspective of youth positive mental health: A critical review. *Br. J. Occup. Ther.* **2015**, *78*, 276–285.
70. Yang, Y.; Li, P.; Fu, X.; Kou, Y. Orientations to happiness and subjective well-being in Chinese adolescents: The roles of prosocial behavior and Internet addictive behavior. *J. Happiness Stud. Interdiscip. Forum Subj. Well-Being* **2017**, *18*, 1747–1762.
71. Lane, D.J.; Mathes, E.W. The pros and cons of having a meaningful life. *Personal. Individ. Differ.* **2018**, *120*, 13–16.
72. Renshaw, T.L.; Long, A.C.J.; Cook, C.R. Assessing adolescents' positive psychological functioning at school: Development and validation of the student subjective wellbeing questionnaire. *Sch. Psychol. Q.* **2015**, *30*, 534–552. https://doi.org/10.1037/spq0000088.
73. Gongora, V.C.; Castro Solano, A. Psychometric properties of the three pathways to well-being scale in a large sample of Argentinean adolescents. *Psychol. Rep.* **2015**, *117*, 167–179. https://doi.org/10.2466/08.02.PR0.117c17z3.
74. Moneta, G.B. The flow model of intrinsic motivation in Chinese: Cultural and personal moderators. *J. Happiness Stud. Interdiscip. Forum Subj. Well-Being* **2004**, *5*, 181–217.
75. Vitterso, J. Subjective well-being versus self-actualization: Using the flow-simplex to promote a conceptual, clarification of subjective quality of life. *Soc. Indic. Res.* **2004**, *65*, 299–331.

76. Rodriguez-Fernandez, A.; Ramos-Diaz, E.; Madariaga, J.M.; Arrivillaga, A.; Galende, N. Steps in the construction and verification of an explanatory model of psychosocial adjustment. *Eur. J. Educ. Psychol.* **2016**, *9*, 20–28.
77. Huo, Y.J.; Binning, K.R.; Molina, L.E. Testing an integrative model of respect: Implications for social engagement and well-being. *Pers. Soc. Psychol. Bull.* **2010**, *36*, 200–212. https://doi.org/10.1177/0146167209356787.
78. Baldwin, C.K.; Caldwell, L.L. Development of the free time motivation scale for adolescents. *J. Leis. Res.* **2003**, *35*, 129–151.
79. Bjorck, J.P.; Kim, G.S.; Cunha, D.A.; Braese, R.W. Assessing religious support in Christian adolescents: Initial validation of the multi-faith religious support scale-adolescent (MFRSS-A). *Psychol. Relig. Spiritual.* **2019**, *11*, 22–31.
80. Arce, E.; Simmons, A.N.; Stein, M.B.; Winkielman, P.; Hitchcock, C.; Paulus, M.P. Association between individual differences in self-reported emotional resilience and the affective perception of neutral faces. *J. Affect. Disord.* **2009**, *114*, 286–293.
81. Archana; Kumar, U.; Singh, R. Resilience and spirituality as predictors of psychological well-being among university students. *J. Psychosoc. Res.* **2014**, *9*, 227–235.
82. Cohn, M.A.; Fredrickson, B.L.; Brown, S.L.; Mikels, J.A.; Conway, A.M. Happiness unpacked: Positive emotions increase life satisfaction by building resilience. *Emotion* **2009**, *9*, 361–368. https://doi.org/10.1037/a0015952.
83. Van Schaick, L.A. Predicting resilience and psychological well-being in early adulthood: The role of religion in childhood and adolescence. *Diss. Abstr. Int. Sect. B Sci. Eng.* **2011**, *71*, 4525.
84. Wright, S.L.; Firsick, D.M.; Kacmarski, J.A.; Jenkins-Guarnieri, M.A. Effects of attachment on coping efficacy, career decision self-efficacy, and life satisfaction. *J. Couns. Dev.* **2017**, *95*, 445–456.
85. Burns, L.R.; D'Zurilla, T.J. Individual differences in perceived information-processing styles in stress and coping situations: development and validation of the perceived modes of processing inventory. *Cogn. Ther. Res.* **1999**, *23*, 345–371.
86. Lau, Y.; Fang, L.; Cheng, L.J.; Kwong, H.K.D. Volunteer motivation, social problem solving, self-efficacy, and mental health: A structural equation model approach. *Educ. Psychol.* **2019**, *39*, 112–132.
87. Benita, M.; Levkovitz, T.; Roth, G. Integrative emotion regulation predicts adolescents' prosocial behavior through the mediation of empathy. *Learn. Instr.* **2017**, *50*, 14–20. https://doi.org/10.1016/j.learninstruc.2016.11.004.
88. Furlong, M.J.; You, S.; Renshaw, T.L.; Smith, D.C.; O'Malley, M.D. Preliminary development and validation of the social and emotional health survey for secondary school students. *Soc. Indic. Res.* **2014**, *117*, 1011–1032.
89. Demyttenaere, K.; Mortier, P.; Kiekens, G.; Bruffaerts, R. Is there enough "interest in and pleasure in" the concept of depression? The development of the Leuven Affect and Pleasure Scale (LAPS). *CNS Spectr.* **2019**, *24*, 265–274. https://doi.org/10.1017/S1092852917000578.
90. Renshaw, T.L.; Olinger Steeves, R.M. What good is gratitude in youth and schools? A systematic review and meta-analysis of correlates and intervention outcomes. *Psychol. Sch.* **2016**, *53*, 286–305.
91. Cheng, S.-T. Endowment and contrast: The role of positive and negative emotions on well-being appraisal. *Personal. Individ. Differ.* **2004**, *37*, 905–915. https://doi.org/10.1016/j.paid.2003.10.013.
92. Beduna, K.; Perrone-McGovern, K.M. Relationships among emotional and intellectual overexcitability, emotional IQ, and subjective well-being. *Roeper Rev.* **2016**, *38*, 24–31. https://doi.org/10.1080/02783193.2015.1112862.
93. Gomez-Baya, D.; Mendoza, R.; Gaspar, T.; Gomes, P. Responses to positive affect, life satisfaction and self-esteem: A cross-lagged panel analysis during middle adolescence. *Scand. J. Psychol.* **2018**, *59*, 462–472. https://doi.org/10.1111/sjop.12450.
94. Shoshana, A. Youth, class, and happiness. *Child. Youth Serv. Rev.* **2019**, *99*, 64–73.
95. Kitayama, S.; Markus, H.R.; Kurokawa, M. Culture, emotion, and well-being: Good feelings in Japan and the United States. *Cogn. Emot.* **2000**, *14*, 93–124. https://doi.org/10.1080/026999300379003.
96. Chinekesh, A.; Hosseini, S.A.; Mohammadi, F.; Motlagh, M.E.; Baradaran Eftekhari, M.; Djalalinia, S.; Ardalan, G. An explanatory model for the concept of mental health in Iranian youth. *F1000Res* **2018**, *7*, 52. https://doi.org/10.12688/f1000research.12893.2.
97. O'Higgins, S.; Sixsmith, J.; Gabhainn, S.N. Adolescents' perceptions of the words "health" and "happy". *Health Educ.* **2010**, *110*, 367–381.
98. Kim-Prieto, C.; Diener, E.; Tamir, M.; Scollon, C.; Diener, M. Integrating the diverse definitions of happiness: A time-sequential framework of subjective well-being. *J. Happiness Stud.* **2005**, *6*, 261–300.
99. Dolan, P.; Kudrna, L. Sentimental hedonism: Pleasure, purpose, and public policy. In *Handbook of Eudaimonic Well-Being*; Springer: New York, NY, USA, 2016; pp. 437–452.
100. Demir, M.; Ozen, A.; Dogan, A. Friendship, perceived mattering and happiness: A study of American and Turkish college students. *J. Soc. Psychol.* **2012**, *152*, 659–664. https://doi.org/10.1080/00224545.2011.650237.

101. Bornstein, R.F.; Huprich, S.K. Construct validity of the relationship profile test: Three-year retest reliability and links with core personality traits, object relations, and interpersonal problems. *J. Personal. Assess.* **2006**, *86*, 162–171. https://doi.org/10.1207/s15327752jpa8602_05.
102. Hatcher, R.L.; Rogers, D.T. Development and validation of a measure of interpersonal strengths: The Inventory of Interpersonal Strengths. *Psychol. Assess.* **2009**, *21*, 554–569. https://doi.org/10.1037/a0017269.
103. Li, T.; Fok, H.K.; Fung, H.H. Is reciprocity always beneficial? Age differences in the association between support balance and life satisfaction. *Aging Ment. Health* **2011**, *15*, 541–547.
104. Kindelberger, C.; Tsao, R. Staying alone or getting attached: Development of the motivations toward romantic relationships during adolescence. *J. Genet. Psychol.* **2014**, *175*, 147–162. https://doi.org/10.1080/00221325.2013.834291.
105. La Guardia, J.G.; Ryan, R.M.; Couchman, C.E.; Deci, E.L. Within-person variation in security of attachment: A self-determination theory perspective on attachment, need fulfillment, and well-being. *J. Pers. Soc. Psychol.* **2000**, *79*, 367–384. https://doi.org/10.1037//0022-3514.79.3.367.
106. Barry, M.B. Special day class students' perceptions of school connectedness. *Diss. Abstr. Int. Sect. A Humanit. Soc. Sci.* **2015**, *76*.
107. Oberle, E.; Schonert-Reichl, K.A.; Zumbo, B.D. Life satisfaction in early adolescence: Personal, neighborhood, school, family, and peer influences. *J. Youth Adolesc.* **2011**, *40*, 889–901. https://doi.org/10.1007/s10964-010-9599-1.
108. Bradley, K.D.; Cunningham, J.D.; Gilman, R. Measuring adolescent life satisfaction: A psychometric investigation of the multidimensional students' life satisfaction scale (MSLSS). *J. Happiness Stud. Interdiscip. Forum Subj. Well-Being* **2014**, *15*, 1333–1345.
109. Gilman, R.; Huebner, S. A review of life satisfaction research with children and adolescents. *Sch. Psychol. Q.* **2003**, *18*, 192–205.
110. Zou, C.; Schimmack, U.; Gere, J. The validity of well-being measures: A multiple-indicator-multiple-rater model. *Psychol. Assess.* **2013**, *25*, 1247–1254. https://doi.org/10.1037/a0033902.
111. Bourke, L.; Geldens, P.M. Subjective wellbeing and its meaning for young people in a rural Australian center. *Soc. Indic. Res.* **2006**, *82*, 165–187. https://doi.org/10.1007/s11205-006-9031-0.
112. Long, R.F.; Huebner, E.S.; Wedell, D.H.; Hills, K.J. Measuring school-related subjective well-being in adolescents. *Am. J. Orthopsychiatry* **2012**, *82*, 50–60. https://doi.org/10.1111/j.1939-0025.2011.01130.x.
113. Palomar-Lever, J.; Victorio-Estrada, A. Determinants of subjective well-being in adolescent children of recipients of the oportunidades human development program in Mexico. *Soc. Indic. Res.* **2013**, *118*, 103–124. https://doi.org/10.1007/s11205-013-0407-7.
114. Wei, M.; Liao, K.Y.-H.; Ku, T.-Y.; Shaffer, P.A. Attachment, self-compassion, empathy, and subjective well-being among college students and community adults. *J. Personal.* **2011**, *79*, 191–221.
115. Bond, L.; Lusher, D.; Williams, I.; Butler, H. Friends or foes? Relational dissonance and adolescent psychological wellbeing. *PLoS ONE* **2014**, *9*, e83388. https://doi.org/10.1371/journal.pone.0083388.
116. Fernandes, H.M.; Vasconcelos-Raposo, J.; Teixeira, C.M. Preliminary analysis of the psychometric properties of Ryff's scales of psychological well-being in Portuguese adolescents. *Span. J. Psychol.* **2010**, *13*, 1032–1043. https://doi.org/10.1017/s1138741600002675.
117. Gao, J.; McLellan, R. Using Ryff's scales of psychological well-being in adolescents in mainland China. *BMC Psychol.* **2018**, *6*, 17. https://doi.org/10.1186/s40359-018-0231-6.
118. Smith, O.R.F.; Melkevik, O.; Samdal, O.; Larsen, T.M.; Haug, E. Psychometric properties of the five-item version of the mindful awareness attention scale (MAAS) in Norwegian adolescents. *Scand. J. Public Health* **2017**, *45*, 373–380. https://doi.org/10.1177/1403494817699321.
119. Renshaw, T.L. Preliminary development and validation of the mindful student questionnaire. *Assess. Eff. Interv.* **2017**, *42*, 168–175.
120. Maltby, J.; Lewis, C.A.; Day, L. Religious orientation and psychological well-being: The role of the frequency of personal prayer. *Br. J. Health Psychol.* **1999**, *4*, 363–378. https://doi.org/10.1348/135910799168704.
121. Caprara, G.V.; Alessandri, G.; Eisenberg, N.; Kupfer, A.; Steca, P.; Caprara, M.G.; Yamaguchi, S.; Fukuzawa, A.; Abela, J. The positivity scale. *Psychol. Assess.* **2012**, *24*, 701–712. https://doi.org/10.1037/a0026681.
122. Morris, C.; Janssens, A.; Shilling, V.; Allard, A.; Fellowes, A.; Tomlinson, R.; Williams, J.; Thompson Coon, J.; Rogers, M.; Beresford, B.; et al. Meaningful health outcomes for paediatric neurodisability: Stakeholder prioritisation and appropriateness of patient reported outcome measures. *Health Qual. Life Outcomes* **2015**, *13*, 87. https://doi.org/10.1186/s12955-015-0284-7.
123. Vaingankar, J.A.; Subramaniam, M.; Chong, S.A.; Abdin, E.; Orlando Edelen, M.; Picco, L.; Lim, Y.W.; Phua, M.Y.; Chua, B.Y.; Tee, J.Y.; et al. The positive mental health instrument: Development and validation of a culturally relevant scale in a multi-ethnic Asian population. *Health Qual. Life Outcomes* **2011**, *9*, 92. https://doi.org/10.1186/1477-7525-9-92.
124. Lin, J.; Chadi, N.; Shrier, L. Mindfulness-based interventions for adolescent health. *Curr. Opin. Pediatrics* **2019**, *31*, 469–475. https://doi.org/10.1097/MOP.0000000000000760.

125. Sukarieh, M.; Tannock, S. The positivity imperative: A critical look at the 'new' youth development movement. *J. Youth Stud.* **2011**, *14*, 675–691. https://doi.org/10.1080/13676261.2011.571663.
126. Hardy, S.A.; King, P.E. Processes of religious and spiritual influence in adolescence: Introduction to a special section. *J. Res. Adolesc. Off. J. Soc. Res. Adolesc.* **2019**, *29*, 244–253. https://doi.org/10.1111/jora.12509.
127. Koenig, H.G. Spirituality and mental health. *Int. J. Appl. Psychoanal. Stud.* **2010**, *7*, 116–122. https://doi.org/10.1002/aps.239.
128. Levin, J. Religion and mental health: Theory and research. *Int. J. Appl. Psychoanal. Stud.* **2010**, *7*, 102–115. https://doi.org/10.1002/aps.240.
129. Hackney, C.H.; Sanders, G.S. Religiosity and mental health: A meta-analysis of recent studies. *J. Sci. Study Relig.* **2003**, *42*, 43–55. https://doi.org/10.1111/1468-5906.t01-1-00160.
130. Muris, P.; Meesters, C.; Pierik, A.; de Kock, B. Good for the self: Self-compassion and other self-related constructs in relation to symptoms of anxiety and depression in non-clinical youths. *J. Child Fam. Stud.* **2016**, *25*, 607–617. https://doi.org/10.1007/s10826-015-0235-2.
131. Muris, P.; Meesters, C.; Timmermans, A. Some youths have a gloomy side: Correlates of the dark triad personality traits in non-clinical adolescents. *Child Psychiatry Hum. Dev.* **2013**, *44*, 658–665. https://doi.org/10.1007/s10578-013-0359-9.
132. Robak, R.W.; Nagda, P.R. Psychological needs: A study of what makes life satisfying. *North Am. J. Psychol.* **2011**, *13*, 75–86.
